# Supplementary material for: Harnessing hybrid perception on multi-scale features for hand-foot-mouth disease multi-region prediction based on Seq2Seq
Source: PLoS One. 2025 Jun 27;20(6):e0326206. doi: 10.1371/journal.pone.0326206 (PMC12204527; doi:10.1371/journal.pone.0326206)
Supplement: Attachment — Submitted filename: Response to Reviewers.pdf [file pone.0326206.s002.pdf]

Response to the Comments from Reviewers

(Manuscript Number: PONE-D-25-08806)

Dear editors and anonymous reviewers:

We sincerely appreciate your thorough review of our manuscript. Your feedback and suggestions have been instrumental in improving the quality of this manuscript. After careful consideration of the suggestions provided by both the editor and reviewers, we have meticulously revised the manuscript.

Table 1 Summary of the pages for editor and each reviewer

Role	Pages
**Response to Journal Requirements**	1-2
**Response to Reviewer #1**	2-5
**Response to Reviewer #2**	5-9
**Response to Reviewer #3**	9-12

Response to Journal Requirements:

1. Please ensure that your manuscript meets PLOS ONE's style requirements, including those for file naming.

Response: Our manuscript was compiled using the provided PLOS ONE LaTeX template. We managed references via BibTeX using the ‘plos2015’ style, incorporating the .bbl output into the .tex file. Figures were formatted according to PLOS ONE’s NAAS recommendations. Tables were constructed using the LaTeX template’s built-in structures. We confirm that these procedures have been followed to meet the journal’s style standards. We confirm that the manuscript adheres to these requirements as described above. Should any unexpected issues be found, please do not hesitate to let us know, and we will be happy to revise accordingly.

2. Please note that PLOS ONE has specific guidelines on code sharing for submissions in which author-generated code underpins the findings in the manuscript. In these cases, we expect all author-generated code to be made available without restrictions upon publication of the work.

Response: The code and data used in this paper have been uploaded to https://github.com/zxj550702/hfmd_predicate.

3. Thank you for stating the following financial disclosure:
This work is funded by:

Bingbing Lei, Scientific research project of Ningxia Education Department(NYG2024084);

Tao Zhou, National Natural Science Foundation of China(62062003).

Please state what role the funders took in the study. If the funders had no role, please state: "The funders had no role in study design, data collection and analysis, decision to publish, or preparation of the manuscript."

If this statement is not correct you must amend it as needed.
Please include this amended Role of Funder statement in your cover letter; we will change the online submission form on your behalf.
Response:The Amended Role of Funder Statement:
Bingbing Lei, Scientific research project of Ningxia Education Department (NYG2024084): This funder supported the study and contributed to the study design through providing ideas for the research framework.
Tao Zhou, National Natural Science Foundation of China (62062003): This funder supported the study and contributed to the data analysis and figure preparation by providing guidance.
The above statement has been added in the coverletter

4. We note you have included a table to which you do not refer in the text of your manuscript. Please ensure that you refer to Table 1 in your text; if accepted, production will need this reference to link the reader to the Table.
Response: Thank you for the correction. In the revised manuscript, we refer to the Table 1 in section Graph convolution neural network. Because this is where a lot of symbols are mentioned.

Response to Reviewer #1:

Concern 1-1: The author lacks a clear statement of the research motivation. In the contribution section, the author directly lists the frequency-enhanced channel attention mechanism, but does not explain why this module was considered beforehand.
Response: Based on your suggestions, we have revised the description of the research motivation in both the abstract and the fourth paragraph of the introduction, making it more concise and precise. Additionally, to supplement the mention of "Furthermore, existing models overlook the utilization of frequency-domain features, which harbor significant information pertinent to the analysis." in the abstract, we have elaborated on the rationale for introducing this mechanism in the fifth paragraph of the introduction.

Abstract
Accurate prediction of Hand, Foot, and Mouth Disease (HFMD) is crucial for effective epidemic prevention and control. Existing prediction models often overlook the cross-regional transmission dynamics of HFMD, limiting their applicability to single regions. Furthermore, their ability to perceive spatio-temporal features holistically remains limited, hindering the precise modeling of epidemic trends. To address these limitations, a novel HFMD prediction model named Seq2Seq-HMF is proposed, which is based on the Sequence-to-Sequence(Seq2Seq) framework. This model leverages hybrid perception of multi-scale features...

Introduction
Paragraph 6
...Nevertheless, the spread of HFMD is a dynamic spatial process, characterized by randomness, uncertainty, and intricate spatio-temporal fluctuations. Traditional prediction methods often struggle to accurately capture the characteristics of multi-regional HFMD epidemic transmission simultaneously. And these methods often overlook the extraction of frequency domain features. Frequency domain features can unveil the periodicity, seasonality, and relative intensity of various frequency components, which are challenging to directly observe in the time domain. These features offer crucial information

that enhances the prediction accuracy of HFMD models. Therefore, it is crucial to develop a methodology that effectively captures nonlinear associations and spatio-temporal dependencies to improve prediction accuracy. Specifically, the core challenge is developing a model that effectively integrates multiple factors, enabling it to capture spatio-temporal dependencies, perform spatio-temporal inference, and account for key influencing variables concurrently. Moreover, simultaneous prediction of epidemic trends across multiple regions, along with multi-step prediction(e.g. , short-, medium-, and long-term), would better support the dynamic allocation of prevention and control resources and the development of emergency plans.

Concern 1-2: The author only discusses time series prediction models in the context of the prediction task for hand, foot, and mouth disease in the related work section. However, we know that time series prediction models are widely used and have a long research history. The author lacks an in-depth review of the relevant work.

Response: Thank you for your detailed addition to the completeness of the related work. In the revised manuscript, we have incorporated a more comprehensive analysis and discussion of the existing literature pertinent to time series prediction models.

Concern 1-3: The section on model structure lacks a clear and easy-to-understand diagram of the model architecture.

Response: Thanks for your valuable suggestion. In response to your suggestion, we modified the model description in the Model Architecture section and provided additional details in the notes for Fig. 1 and Fig. 2.

Model architecture

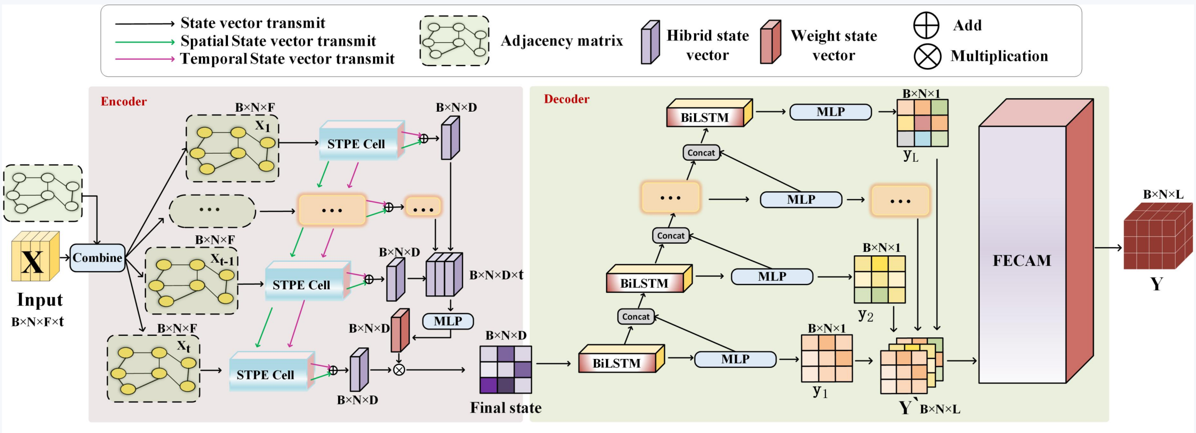

Fig. 1 The overall structure of Seq2Seq-HMF. It comprises an encoder and a decoder. The encoder performs multi-scale hibrid perception on input time series data from multiple regions to extract features, which the decoder then processes to generate predictive time series data for those regions.

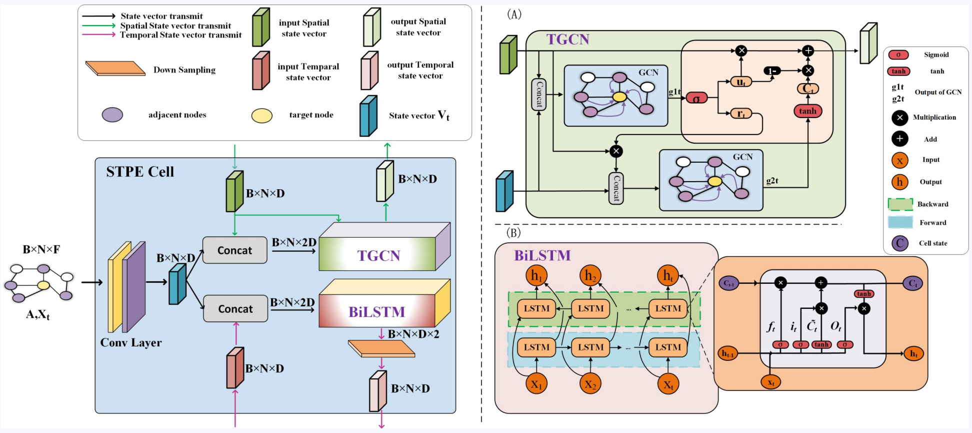

Fig. 2 Structure of STPE Cell. It is contains TGCN (A) and BiLSTM (B). Both modules receive the output of the previous STPE Cell in addition to the data features.

Concern 1-4: The experimental results section lacks a detailed description of the dataset and the data preprocessing methods.

Response: Thanks for your valuable feedback. In response to your suggestion, we have relocated the data collection section from the Methods section to the Experiments section. Additionally, we have enriched this section with description of the data preprocessing methods employed.

Concern 1-5: In the experimental results section, the author does not provide an introduction to the baseline models, instead placing some of this information in the introduction section, which seems inappropriate. Additionally, all the baseline models listed by the author are deep learning models, lacking comparisons with classical hand, foot, and mouth disease prediction models and traditional machine learning models. It is recommended to include these comparisons.

Response: Thanks for your constructive comments. According to your comments, we added the description of the baseline model in the comparison test section, and added the Random Forest Regression(RFR) and Extreme Gradient Boosting(XGBoost) models in the machine learning model into the experiment as comparison experiments. Based on the experimental results, Table 3, Figure 4, Figure 5 and Figure 6 were modified.

Concern 1-6: The experimental results section only analyzes the individual experiments and lacks an overall summary of the experimental results.

Response: Thanks for your valuable feedback. In the revised manuscript, the conclusion section has been extended to improve clarity by providing a more detailed summary of the experimental results.

Conclusion

Paragraph 1

This study proposed a prediction model of HFMD, Seq2Seq-HMF, which performs a multi-region multi-step prediction task. The model consists of an encoder based on STPE cell and a decoder that incorporates FECAM. In the experimental section, the performance of eight HFMD prediction models was evaluated in multi-region and multi-step prediction tasks, using Japan's 47 prefectures and a Chinese city as a case study. Among these models, the Seq2Seq-HMF model demonstrated higher accuracy in predicting the number of HFMD cases for the upcoming weeks and exhibited greater

precision and stability in both short- and long-term predictions.

Concern 1-7: The model should be validated on multiple datasets.

Response: We appreciate the reviewer's suggestion regarding the validation of our model on multiple datasets. We have addressed this concern by evaluating our proposed Seq2Seq-HMF model using diverse datasets, as detailed below:

Multi-Region Dataset (Japan): The primary dataset used for evaluating the multi-region, multi-step prediction capability of Seq2Seq-HMF consists of weekly Hand, Foot, and Mouth Disease (HFMD) case counts from December 2013 to December 2023 for all 47 prefectures of Japan. This data, obtained from the National Institute for Infectious Diseases (NIID), was complemented with corresponding weekly meteorological data (temperature, relative humidity, atmospheric pressure, wind speed, and rainfall) sourced from the Japan Meteorological Agency (JMA). This comprehensive multi-source dataset allows us to assess the model's ability to capture spatiotemporal dependencies across different geographical areas.

Single-Region Dataset (China): To provide a different perspective on the model's performance and its generalizability to different data granularities and geographical contexts, we additionally utilized a single-region dataset. This dataset comprises daily HFMD case counts and meteorological variables for a specific city in southern China, spanning from January 2014 to December 2019. This publicly available dataset, released by the China National Center for Disease Control and Prevention, allows us to evaluate the model's performance on daily data and in a different epidemiological setting.

By validating Seq2Seq-HMF on both a multi-regional, weekly dataset from Japan and a single-regional, daily dataset from China, we demonstrate the model's effectiveness across different spatial scopes, temporal resolutions, and geographical locations.

Response to Reviewer #2:

Overall, the author manages to clearly convey their work, especially the designed DL framework. However, there are still aspects that need improvement. Specifically, the accuracy of vocabulary and grammar usage requires attention. Regarding the dataset, it is rather simplistic. For instance, the data from Chengdu mentioned in Reference 21 could be incorporated for experiments and comparisons. This would enhance the comprehensiveness and validity of the research. Finally, the author is advised to explore and incorporate more SOTA DL methods. This would not only bring novelty to the study but also potentially lead to more accurate and efficient results in predicting HFMD cases.

Response: Thank you for your positive feedback on our manuscript. We have provided point-by-point responses to your concerns, and we hope they address any remaining issues. In addition, we have thoroughly revisited certain parts of the paper in light of your comments and made enrichments and revisions accordingly.

Concern 2-1: Please provide the links to the data and code.

Response: The code and data used in this paper have been uploaded to https://github.com/zxj550702/hfmd_predicate.

Concern 2-2: The abstract contains some errors and inappropriate places, please revise carefully,

such as:

a. there are some grammar errors in the abstract, please check carefully.(also in the main body of this manuscript)

b. some descriptions in the abstract are unclear. For example: ‘significant information pertinent to the analysis.’

c. why is the title of the paper included in the abstract?

Response: Thank you for pointing out these specific areas for improvement in the abstract. We have carefully reviewed your comments regarding grammatical errors, unclear descriptions, and the inclusion of the paper title. We have revised the abstract accordingly to correct these issues, improve clarity, and ensure it is concise and informative. We have also checked the main manuscript body for similar grammatical errors and made necessary corrections throughout. All changes, including these revisions to the abstract, are clearly marked in the file ‘Revised Manuscript with Track Changes’ for your review.

Concern 2-3: Please correct the use of proprietary terms and abbreviations, such as SVR.

Response: Thank you for the details. In the revised manuscript, “Support Vector Regression(SVR)” was explained in introduction section. And we have proofread more of the use of proprietary terms and abbreviations to make them accurate.

Concern 2-4: Lines 34-42 of the main text are logically disorganized, with unclear sentence structure and poor consistency in word usage.

Response: Thank you for the details. In the revised manuscript, Lines 34-42 of the main text have been revised to address issues of confused logic, unclear sentence structure, and poor word consistency.

Concern 2-5: Why not attempt to test and compare the data of Chengdu city on this dataset, as mentioned in reference 21?

Response: We appreciate the suggestion to test our model on the Chengdu city data mentioned in reference 21. Regarding this specific dataset, we found that it is not publicly available, which limits its direct use for validation in this study. Through our efforts to obtain diverse data for comprehensive validation, we acquired the following datasets, as detailed below:

This dataset comprises daily HFMD case counts and meteorological variables for a specific city in southern China, spanning from January 2014 to December 2019. This publicly available dataset, released by the China National Center for Disease Control and Prevention, allows us to evaluate the model’s performance on daily data and in a different epidemiological setting.

Concern 2-6: After introducing the three main contributions of the Seq2Seq-HMF model, the results of the comparative experiments and ablation experiments are directly mentioned, lacking a brief explanation of the experimental setup and objectives. It would be beneficial to supplement that these experiments were conducted to verify the effectiveness of the model and the functions of each module, enabling readers to better understand the significance of the experimental results.

Response: Thank you for your constructive comments. We appreciate the suggestion to clarify the experimental setup and objectives. In the revised manuscript, these details have been explained in the ‘Experimental Setup’ section. This section now clearly outlines the purpose of the comparative and ablation experiments, which were designed to verify the overall effectiveness of the Seq2Seq-HMF model and the specific functions of its individual modules, thereby enhancing

the reader’s understanding of the subsequent results.

Experimental setup

Paragraph 2

Comparative experiments are conducted respectively on multi-region and single-region datasets. In the multi-region dataset, 47 counties were ranked based on official data from Japan. The goal of these experiments was to compare the prediction effects of the RFR, XGBoost, TGCN, STGCN, LSTM, Seq2Seq-Shil, Seq2Seq-HMF, and DA-RNN models and to verify the validity of the Seq2Seq-HMF model. Otherwise, to clarify the impact of each component on Seq2Seq-HMF performance, the ablation study section systematically evaluates the key components of the model through ablation experiments and analyzes the experimental results on multi-region dataset.

Concern 2-7: Table 1 has not been explained in the main text. If it is necessary to list the symbols used in the text, please choose an appropriate way.

Response: Thanks for your valuable suggestion. In the revised manuscript, we refer to the symbol table in section Graph convolution neural network. Because this is where a lot of symbols are mentioned.

Concern 2-8: Since your method does not involve GNNs, I believe that there is no need for you to provide a detailed description of GNNs in the main text. A simple description is sufficient, just like the descriptions of other methods in the above text. If you want to facilitate the readers' understanding, you can choose to place the relevant content in the sup.

Response: Response: Thank you for your valuable suggestion. We agree that since GNNs are not a central component of our proposed method, a detailed description in the main text is not necessary. In the revised manuscript, we have simplified the description of GNNs, in line with your suggestion, to streamline the main text and allow readers to focus on the core contributions of our method.

Graph convolution neural network

Graph Convolution Networks (GCN) were proposed as a solution to the expensive computational costs of GNN...

Concern 2-9. Why are the "Evaluation metrics" placed in the "Experimental results" chapter?

Response: Thank you for the details. Given that the evaluation metric constitutes the foundation for evaluating model performance, we have incorporated it into the final section of the Methods chapter in the revised manuscript.

Concern 2-10. In line 295, you mentioned "a state vector depth of D = 128". Does this refer to the hidden layer parameter of the LSTM or some other parameter? Please provide a detailed explanation. Since the number of layers in a recurrent neural network generally should not be too large, an excessively large number of layers can lead to problems with gradient propagation.

Response: Thanks for your valuable suggestion. We are very sorry for the confusion caused by our unclear expression. In the revised manuscript, we have corrected the expression. The number of neurons is described here because we would to show the reader some important parameter settings in the model. When the reader understands these parameters, the model structure shown

in Fig. 1 could be understood more quickly.

Experimental setup

Paragraph 1

... Given the model architecture wherein each layer consists of D=128 neurons, it is imperative to adjust the state vector accordingly to ensure compatibility with the specified neural network structure...

Concern 2-11.Figure 5 looks rather chaotic. The information it contains has not been reasonably compressed but is merely presented in an intuitive manner. Please highlight the information you intend to convey. For multiple variables, you can use evaluation metrics for comparison, or display them separately and emphasize their excellent performance.

Response: Thanks for your valuable suggestion. To facilitate an intuitive comparison of predicted and actual values, we generated Figure 5. However, the inclusion of multiple models within a single figure resulted in a cluttered and confusing presentation. Consequently, we have refined Figure 5 to showcase only one representative model per category. Meanwhile, the description in Figure 5 has been explained in more detail in the revised manuscript to make it easier to understand. Specific modifications are as follows:

Comparison results and analysis

Paragraph 3

...To directly compare model performance, this paper presents the prediction outcomes for four representative models at L=1. Fig 5 respectively shows the comparison between the predicted value and the measured value of different network models when the prediction step size $L=1$, where the abscess Node number represents the number of city nodes, the ordinate Week represents the time series from week 1 to week 110 of the test set, and the vertical coordinate represents the value of HFMD cases. It indicates that the prediction outcomes of STGCN models is less than optimal. Specifically, during the peak period of HFMD incidence, STGCN tend to overemphasize the influence of neighboring nodes. This leads to low-incidence nodes being inappropriately affected by adjacent high-incidence nodes, resulting in a notably reduced prediction accuracy. In contrast, the DA-RNN model, all based on RNNs, have achieved higher accuracy in predicting HFMD. Particularly, the Seq2Seq-HMF model demonstrates the closest alignment between predicted results and actual values during peak incidence periods.

Concern 2-12. In Figures 6 and 7, all the lines are mixed together, making it impossible to observe useful information. Additionally, in these figures, each color represents the prediction results of a fixed model. Why not just have one legend? It seems that your figures were drawn using Python. Please integrate all the legends. In the Python code, only one plot should have the ‘plt.legend’ set.

Response: We appreciate your feedback on Figures 6 and 7. As suggested, we have revised these figures in the revised manuscript. Initially, each individual graph was created separately and combined, resulting in multiple legends. In the revised version, we have integrated all plots into a single figure with a unified legend, as you recommended. This streamlines the presentation and enhances clarity. In addition, for improved clarity, we have limited the number of models presented in the figures to four, down from the original eight.

Concern 2-13.In Figure 8, you might consider placing different evaluation metrics in separate

graphs. Also, the form of "R2" is not reasonable. You can manually modify the text description in the graph and express R square correctly using a mathematical expression.

Response: Response: Thank you for your valuable feedback on the presentation of our evaluation metrics. In response to your suggestion, we have revised Fig 8(as Fig 9 in revised manuscript) to place different evaluation metrics in separate graphs to enhance clarity. We believe these changes improve the readability and accuracy of the results presentation.

Fig 9:

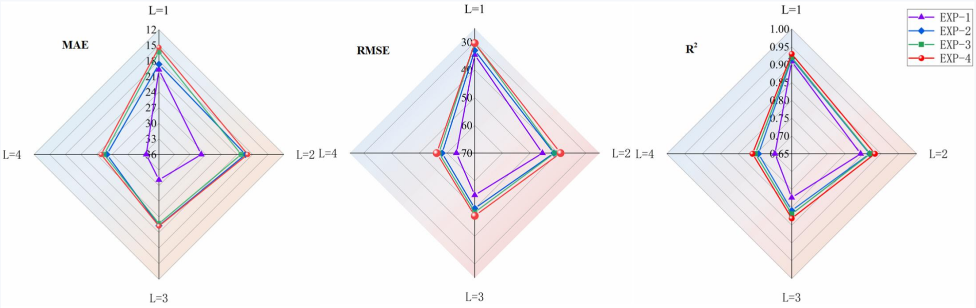

Response to Reviewer #3:

The manuscript PONE-D-25-08806 proposes a Seq2Seq-based deep learning model for predicting the spatial dynamics of hand, foot, and mouth disease (HFMD). While the model architecture appears innovative, the following points require clarification and improvement:

Response: Thank you for your positive feedback on our manuscript. We have tried our best to address your concerns with detailed,point-by-point responses. Furthermore, we have carefully revisited specific sections of the paper based on your suggestions and have made comprehensive revisions and improvements accordingly.

Concern 3-1: The title and introduction emphasize multi-region HFMD prediction as a key contribution. However, Figures 6 and 7 focus on single-region case predictions. How does simultaneous multi-region prediction fundamentally differ from separate single-region modeling? How is the claimed spatiotemporal dependency explicitly demonstrated in the current experiments? Visualizing spatial interaction patterns (e.g., using ArcGIS) to compare multi-region and single-region predictions would strengthen this claim.

Response: We sincerely appreciate your valuable and critical question. We would like to explain that Figures 6 and 7 are not single-region predictions, but rather visualizations of multi-region predictions, providing a more intuitive complement to Figure 5. Because 47 regions are too many to display, we chose 8 representative regions to visually display and compare the predictions of various models. In the revised manuscript, we have made more concise revisions to this part.

In single-region modeling, models are trained independently for the time series data of each region. This approach not only complicates the prediction process but also causes the model to focus solely on the characteristics of individual regions, potentially overlooking the spatial trends of disease spread in the real world. In contrast, a multi-task architecture allows for the sharing of features between spatially correlated regions, thereby capturing the interdependencies and influences among them. Therefore, we believe that a multi-task architecture provides a more

scientific and comprehensive approach than single-region modeling for multi-region prediction tasks.

In the model structure, the encoder is designed to capture the persistent mutual influence between nodes representing regions. We describe in detail how we extract temporal and spatial dependencies to understand disease transmission trends. Subsequently, in ablation experiments, we demonstrate the importance of the STPE in capturing spatiotemporal dependencies. The results show that modeling these dependencies improves prediction accuracy.

Finally, following your helpful suggestions, we performed a spatial interactive visualization of the predicted results compared with the observed values. This visualization could be found in Fig 8 of the revised manuscript.

Fig 8:

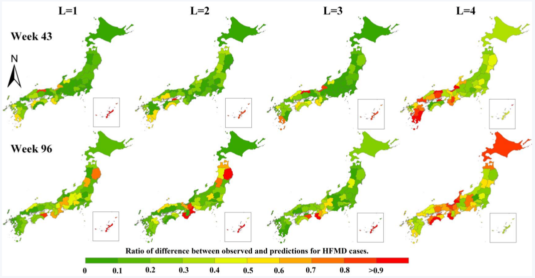

Concern 3-2: Given that multi-region joint prediction could theoretically be achieved through conventional multi-task learning, please address: What specific advantages does the proposed complex architecture offer over simpler multi-task learning frameworks? Is the reported performance improvement statistically significant compared to baseline multi-task approaches?

Response: Thanks for your comment. What we would explained is, when working with spatiotemporal data, complex architectures can learn long-distance dependencies and interaction patterns between different regions, while simple multitask learning frameworks, due to limited shared representations, may only capture local or surface features. At the same time, in the multi-region prediction task, the complex architecture can assign weights adaptively according to the correlation between different regions, so that the model can make full use of the information of other regions when predicting one region. Therefore, the graph convolution operation we set up in the proposed model provides the above effect. In addition, the model has been tested for times to ensure the stability of its performance.

Concern 3-3: While the model emphasizes generalization and accuracy, interpretability is critical for epidemiological applications. Please discuss: How does Seq2Seq-HMF provide insights into HFMD transmission drivers? Are there mechanisms to trace how spatiotemporal dependencies influence predictions?

Response: We sincerely thank the reviewer for their valuable comments and insightful suggestion regarding the importance of interpretability in epidemiological models. We fully agree that understanding the drivers of predictions and the underlying spatiotemporal dependencies is crucial for practical public health applications, beyond just achieving high accuracy and generalization. We acknowledge that the primary focus of the proposed Seq2Seq-HMF model, as presented in the original manuscript, was on enhancing prediction performance through its hybrid feature perception and Seq2Seq framework. Currently, the Seq2Seq-HMF model primarily focuses on learning complex spatiotemporal dependencies from multi-regional, multi-scale data

through its graph-based structure and iterative convolution operations to achieve high-precision predictions. Although the mode's design, such as the graph structure capturing inter-regional relationships and the convolution operations processing spatiotemporal information, implicitly encodes potential drivers within the data (e.g., influences from neighboring regions, historical trends), it does not inherently provide mechanisms for directly quantifying and explaining these drivers. the model itself does not inherently possess a mechanism to directly track and visualize how these specific dependencies influence the final prediction outcome.

In response to this important point, we have revised the Conclusion section of the manuscript. We have added a dedicated sentences discussing the need for interpretability in this context and outlining potential future directions to address this aspect. Thank you again for this constructive feedback, which has helped us clarify the scope and future potential of the work.

Concern 3-4: Were input features standardized/normalized to mitigate bias? How were missing values or outliers handled? Could these methods inadvertently suppress long-term trends in the data?

Response: Thank you for your valuable comments. In this paper, the Min-Max Normalization method is used to normalize the data. A linear interpolation method is applied to the default values. The outliers are not processed, and we expect the model to handle the abnormal data by itself according to the long-term spatiotemporal dependence.

For normalization, Min-Max Normalization scales these features to the same range, making the model easier to work with. Moreover, increases and decreases in cases are usually non-linear and do not suppress long-term trends. For missing values, the dataset provides a very comprehensive set of data, with only one region missing 53 items for one meteorological factor, accounting for only 0.002% of the entire dataset. Therefore, this operation does not suppress the long-term trend of the data.

Concern 3-5: HFMD exhibits seasonal patterns. Was periodicity explicitly modeled (e.g., via month/season encoding)? How sensitive are the results to the spatial weight matrix (e.g., adjacency-based vs. distance/population-mobility-based)? Have alternative spatial weighting schemes been tested for robustness?

Response: Thanks for your constructive comments. We would like to add that the seasonality of HFMD has been explicitly incorporated into the model. The features in this study encompass the week number, providing temporal resolution at a weekly scale. This note was added in the data collection section of the revised manuscript.

Regarding how sensitive are the results to the spatial weight matrix, following your comments, we conducted an ablation experiment on the distance-based spatial weight matrix. Both spatial weight matrix generally capture the extensive spatio-temporal dependence of HFMD propagation, thus improving prediction accuracy. The results indicate that the proximity-based spatial weight matrix outperforms the distance-based one in terms of prediction accuracy for HFMD propagation. This may be because disease spread prediction tasks are more sensitive to the characteristics of neighboring regions, and the distance-based weight matrix may not effectively capture this local dependency. The distance-based spatial weight matrix is less effective, possibly because it fails to fully utilize the strong correlation between neighboring regions and introduces noise from distant units. In contrast, the adjacency-based weight matrix can better capture local dependencies, thus performing better in spatio-temporal feature extraction. Alternative spatial weighting schemes have been tested for robustness to ensure the reliability of model estimates.

Concern 3-6: The design of STPE encoder cells and FECAM decoder modules is presented as novel. What existing methodologies inspired these components? Are there foundational references that support the architectural choices? Citations should be provided.

Response: Thanks for your comments. For STPE, the model structure section makes references to some existing components within it. This demonstrates how the STPE encoder builds upon established methodologies while incorporating innovative elements to address the specific requirements of our task. For FECAM, The introduction section provides further motivation for the inclusion of the FECAM module, which is based on the work proposed in paper[26]. We modified the original architecture to address the unique challenges presented by our study. Specifically as follows:

Introduction

Paragraph 6

(2) Traditional prediction methods often struggle to accurately capture the characteristics of multi-regional HFMD epidemic transmission simultaneously. And these methods often overlook the extraction of frequency domain features. Frequency domain features can unveil the periodicity, seasonality, and relative intensity of various frequency components, which are challenging to directly observe in the time domain. These features offer crucial information that enhances the prediction accuracy of HFMD models. Therefore, it is crucial to develop a methodology that effectively captures nonlinear associations and spatio-temporal dependencies to improve prediction accuracy. Specifically, the core challenge is developing a model that effectively integrates multiple factors, enabling it to capture spatio-temporal dependencies, perform spatio-temporal inference, and account for key influencing variables concurrently. Moreover, simultaneous prediction of epidemic trends across multiple regions, along with multi-step prediction(e.g. , short-, medium-, and long-term), would better support the dynamic allocation of prevention and control resources and the development of emergency plans.

...

(3) A frequency-enhanced Channel Attention mechanism (FECAM) for HFMD prediction is introduced\cite{26}. The FECAM module models the frequency correlation between channels based on discrete cosine transform, which improves the ability of the model to extract frequency features, thus enhancing the accuracy of multi-step prediction.

Model architecture

Encoder with Spatial-Temporal Parallel Encoding Cells（STPE Cell）

Paragraph 1

As shown in Fig 2, each STPE Cell contains a temporal graph convolutional network(TGCN)[35] and a bidirectional long short-term memory network (BiLSTM)...

We believe that these citations and explanations adequately ground our architectural choices in existing methodologies, while also highlighting the innovative adaptations we have implemented. We are happy to provide further clarification or additional citations if needed.

Concern 3-7: All figures require resolution enhancement to ensure readability of labels, legends, and annotations.

Response: Thank you for the details. In the revised manuscript, the resolution of the various images has been carefully adjusted to ensure that they are legible.
