# Supplementary material for: Harnessing hybrid perception on multi-scale features for hand-foot-mouth disease multi-region prediction based on Seq2Seq
Source: PLoS One. 2025 Jun 27;20(6):e0326206. doi: 10.1371/journal.pone.0326206 (PMC12204527; doi:10.1371/journal.pone.0326206)
Supplement: Attachment — Submitted filename: review.docx [file pone.0326206.s001.docx]

1、The author lacks a clear statement of the research motivation. In the contribution section, the author directly lists the frequency-enhanced channel attention mechanism, but does not explain why this module was considered beforehand.

2、The author only discusses time series prediction models in the context of the prediction task for hand, foot, and mouth disease in the related work section. However, we know that time series prediction models are widely used and have a long research history. The author lacks an in-depth review of the relevant work.

3、The section on model structure lacks a clear and easy-to-understand diagram of the model architecture.

4、The experimental results section lacks a detailed description of the dataset and the data preprocessing methods.

5、In the experimental results section, the author does not provide an introduction to the baseline models, instead placing some of this information in the introduction section, which seems inappropriate. Additionally, all the baseline models listed by the author are deep learning models, lacking comparisons with classical hand, foot, and mouth disease prediction models and traditional machine learning models. It is recommended to include these comparisons.

6、The experimental results section only analyzes the individual experiments and lacks an overall summary of the experimental results.

7、The model should be validated on multiple datasets.
